# Supplementary material for: Potential impact of introducing vaccines against COVID-19 under supply and uptake constraints in France: A modelling study
Source: PLoS One. 2021 Apr 28;16(4):e0250797. doi: 10.1371/journal.pone.0250797 (PMC8081204; doi:10.1371/journal.pone.0250797)
Supplement: S1 File — (DOCX) [file pone.0250797.s001.docx]

**Potential impact of introducing vaccines against COVID-19 under supply and uptake constraints in France: a modelling study**

**L Coudeville, O Jollivet, C Mahé, SS Chaves, GB Gomez**
S1 File: S1 Text

# Model overview and structure

## Model design

We expanded a previously published age-structured compartmental transmission model (1) to account for the protection conferred by future vaccination against infection or reduced severity of symptomatic disease.

For each age class, a total of 11 compartments were considered to represent the infection process. A flow diagram of the model structure is provided in S1 Fig.

S1 Fig. Flow diagram of the model


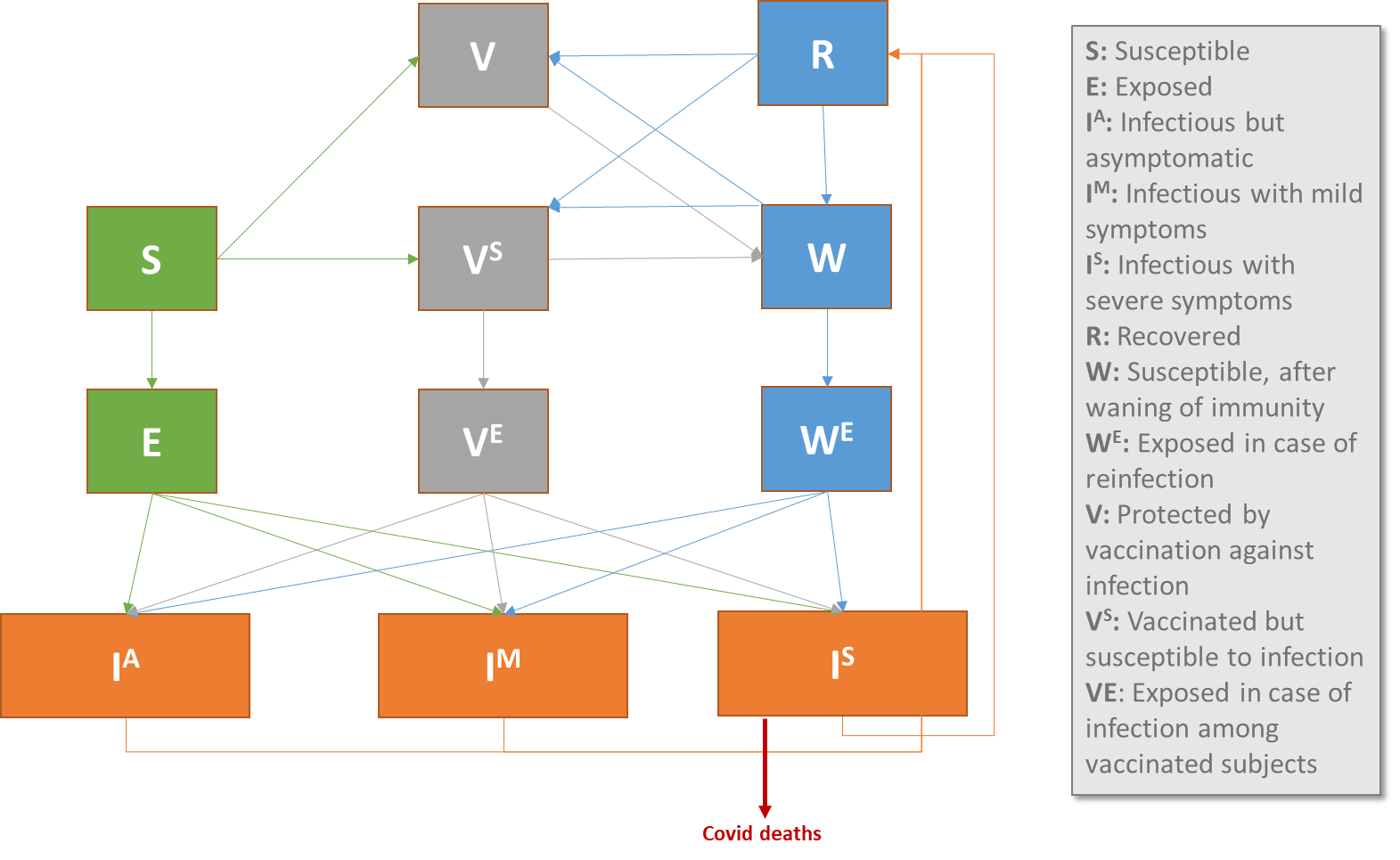


$S$ : Susceptible; $E$ : Exposed; $I^{A}$ : Infectious and asymptomatic; $I^{M}$ : Infectious with mild symptoms; $I^{S}$ : Infectious with severe symptoms; $R$ : Recovered; $W$ : Susceptible after waning of natural immunity; $W^{E}$ : Exposed after waning of natural immunity; $V$ : Protected by vaccination against infection; $V^{S}$ : Partially protected by vaccination and still susceptible to infection; $V^{E}$ : Exposed, having previously been vaccinated at least once.

In the analyses performed, we considered that infections with severe symptoms ($I^{S}$) require hospitalizations and deaths were modelled as a fraction of these severe infections. The set of differential equations that defines the model provides a representation of both the infection process and the demographic processes. Following Hethcote (2), we consider a population with a continuous flow of individuals between age groups. The differential equation that defines the transition from/to compartment $X_{i}$ is given by:

$$\frac{dX_{i,t}}{dt}=D_{i,t}^{X}+I_{i,t}^{X}$$

Where $D_{i,t}^{X}$ and $I_{i,t}^{X}$ correspond respectively to the demographic process and infection process for compartment $X_{i}$ i.e. transition rates between compartments at a given time $t$ due to birth, death or ageing or change in disease status.

### Demographic process

The demographic process is the same across all disease stages and similar to the previous model. For every age group except the first one, it is given by:

$$D_{i,t}^{X}=\Theta_{i-1}X_{i-1,t}-\left( \mu_{i}+\Theta_{i} \right)X_{i,t} \left( i>1 \right)$$

$\mu_{i}$ is the death rate and $\Theta_{i}$ is the transition rate from age group $i$ to age group $i+1$, defined as follows:

$$\Theta_{i}=\frac{\mu_{i}+g}{e^{\left( \mu_{i}+g \right)l_{i}}-1}$$

where g stands for the growth rate of the population and $l_{i}$ for the range in age spanned by age class $i$.

In the first age group, except for the compartment corresponding to disease status at birth, the demographic process simplifies to:

$$D_{1,t}^{X}=\left( \mu_{1}+\Theta_{1} \right)X_{1,t}$$

We consider that all newborns are susceptible. Therefore:

$$D_{1,t}^{S}=B_{t}-\left( \mu_{1}+\Theta_{1} \right)X_{1,t}$$

where $B_{t}$ is the number of births at date $t$ defined as a fraction of the population size.

### Infection process

The infection process is described by the following set of equations:

$$\left\{ \begin{matrix} \begin{matrix} I_{i,t}^{S} & =-\lambda_{i,t}S_{i,t}-v_{i,t}(1-e^{sf}-e^{sp})S_{i,t} \end{matrix} \\ \begin{matrix} I_{i,t}^{E} & =\lambda_{i,t}S_{i,t}-\sigma E_{i,t} \end{matrix} \\ \begin{matrix} \begin{matrix} I_{i,t}^{I^{A}} & =\sigma\left( p_{i}^{sa}E_{i,t}+p_{i}^{va}V_{i,t}^{E}+p_{i}^{wa}W_{i,t}^{E} \right)-\gamma I_{i,t}^{A} \end{matrix} \\ \begin{matrix} I_{i,t}^{I^{M}} & =\sigma\left( p_{i}^{sm}E_{i,t}+p_{i}^{vm}V_{i,t}^{E}+p_{i}^{wm}W_{i,t}^{E} \right)-\gamma I_{i,t}^{M} \end{matrix} \\ \begin{matrix} \begin{matrix} I_{i,t}^{I_{s}} & =\sigma\left( p_{i}^{ss}E_{i,t}+p_{i}^{vs}V_{i,t}^{E}+p_{i}^{ws}W_{i,t}^{E} \right)-\gamma I_{i,t}^{S} \end{matrix} \\ \begin{matrix} I_{i,t}^{R} & =\gamma\left( I_{i,t}^{A}+I_{i,t}^{M}+I_{i,t}^{S} \right)-{(\tau}_{r}+v_{i,t}(1-e^{rf}-e^{rp}))R_{i,t} \end{matrix} \\ \begin{matrix} \begin{matrix} I_{i,t}^{W} & =\tau_{r}R_{i,t}+\tau_{v}\left( V_{i,t}{+V}_{i,t}^{S} \right)-{(\lambda}_{i,t}+v_{i,t}(1-e^{wf}-e^{wp}))W_{i,t} \end{matrix} \\ \begin{matrix} I_{i,t}^{W^{E}} & =\lambda_{i,t}W_{i,t}-\sigma W_{i,t}^{E} \end{matrix} \\ \begin{matrix} \begin{matrix} I_{i,t}^{V} & =v_{i,t}\left( e^{sf}S_{i,t}+{e^{rf}R}_{i,t}+e^{wf}W_{i,t} \right)-\tau_{v}V_{i,t} \end{matrix} \\ \begin{matrix} I_{i,t}^{V^{S}} & =v_{i,t}\left( e^{sp}S_{i,t}+{e^{rp}R}_{i,t}+{e^{wp}W}_{i,t} \right)-\left( {(1-e^{i})\lambda}_{i,t}+\tau_{v} \right)V_{i,t}^{S} \end{matrix} \\ \begin{matrix} I_{i,t}^{V^{E}} & ={(1-e^{i})\lambda}_{i,t}V_{i,t}^{S}-\sigma V_{i,t}^{E} \end{matrix} \end{matrix} \end{matrix} \end{matrix} \end{matrix} \end{matrix} \right.$$

where

- $\sigma$ = rate at which exposed cases become infectious
- $\gamma$ = recovery rate of infectious
- $\tau_{r}$ = waning rate of natural immunity
- $p_{i}^{\mathrm{sa}},p_{i}^{\mathrm{sm}},p_{i}^{\mathrm{ss}}$ = the age-specific probability to develop asymptomatic, mild or severe disease for susceptible individuals, with $p_{i}^{\mathrm{sa}}+ p_{i}^{\mathrm{sm}}+p_{i}^{\mathrm{ss}}=1$
- $p_{i}^{\mathrm{wa}},p_{i}^{\mathrm{wm}},p_{i}^{\mathrm{ws}}$ = the age-specific probability to develop asymptomatic, mild or severe disease in case of reinfection, with $p_{i}^{\mathrm{wa}}+p_{i}^{\mathrm{wm}}+p_{i}^{\mathrm{ws}}=1$
- $p_{i}^{\mathrm{va}},p_{i}^{\mathrm{vm}},p_{i}^{\mathrm{vs}}$ = the age-specific probability to develop asymptomatic, mild or severe disease in case of vaccine failure, with $p_{i}^{\mathrm{va}}+p_{i}^{\mathrm{vm}}+p_{i}^{\mathrm{vs}}=1$
- $v_{i,t}$ = rate at which population of age i is vaccinated at time t
- $e^{sf},e^{rf}, e^{wf}$ = ability of vaccination to provide full protection for individuals in state S, R or W prior to vaccination
- $e^{sp},e^{rp}, e^{wp}$ = ability of vaccination to provide partial protection for individuals in state S, R or W prior to vaccination with $e^{sf}+e^{sp}\leq1,e^{rf}+e^{rp}\leq1,e^{wf}+e^{wp}\leq1$
- $e^{i}$ = ability of vaccination to reduce infection risk in case of exposure to infectious individuals
- $\tau_{v}$ = waning rate of vaccine-conferred immunity

In this representation, vaccination has three potential impacts:

1. It can provide full protection against infection for a limited duration. This ability depends on the status of the subject before vaccination (Susceptible, Recovered or Susceptible after recovery from an infection). Please note that we exclude the situation where an individual loses his naturally acquired protection after vaccination.
2. It can reduce the susceptibility to infection for a limited duration.
3. It can reduce the severity of symptoms in case of infection.

It is worth mentioning that under this representation vaccination has no direct impact when vaccine is administered to an individual currently developing an infection.

The force of infection is given by:

$$\lambda_{i,t}=\beta d_{t}s_{t}\frac{\sum_{j=1}^{n} \beta c_{ij}\left( I_{i,t}^{S}+I_{i,t}^{M}+\alpha I_{i,t}^{A} \right)}{\sum_{j=1}^{n} P_{j}}+\epsilon_{t}$$

where

- $\beta$ = transmission rate between an infectious and a susceptible individual
- $\alpha$ = reduction factor of infectiousness for asymptomatic cases
- $c_{\mathrm{ij}}$ = contact rate between individuals of age $i$ and $j$ at risk of disease transmission
- $\epsilon_{t}$ = force of infection due to imported case at date $t$
- $d_{t}$ = reduction factor related to control measures implemented at date $t$
- $P_{j}$ = population size in age group $j$

The model was developed in R 3.6.0 (3) and RStudio (4) using the deSolve package (5). To simplify the analysis, we considered a standard month duration of 30 days in the simulations.

## Model parameterisation

We used the example of France as a case study for assessing the potential impact of vaccination. Population size, age structure, background mortality, and birth rates were sourced from the United Nations Population Division (6). Background mortality and birth rate were held constant in the analysis. In addition to demographic information, we also used French-specific information such as social contact matrices by age (7). Parameters related to COVID-19 infection process are presented in S1 Table .

**S1 Table.** Parameters related to COVID-19 infection process.

| **Parameter** | **Baseline [range explored]** | **Reference** |
| --- | --- | --- |
| Infection-fatality ratio % | 0-19y: 0.001 [0-0.002]  20-29y: 0.007 [0.003-0.01]  30-39y: 0.02[0.01-0.04]  40-49y: 0.06 [0.03-0.09]  50-59y: 0.2 [0.1-0.36]  60-69y: 0.9 [0.5-1.4]  70-79y: 2.4 [1.4-3.7]  80yr+: 10.1 [6.0-15.6] | Salje et al.(8) |
| Infections requiring hospitalization, % | 0-19y: 0.2 [0.1-0.3]  20-29y: 0.6 [0.4-1.0]  30-39y: 1.3 [0.8-2.0]  40-49y: 1.7 [1.0-2.7]  50-59y: 3.5 [2.1-5.4]  60-69y: 7.1 [4.2-11.0]  70-79y: 11.3 [6.7-17.5]  80yr+: 32.0 [19.0-49.4] | Salje et al.(8) |
| % of symptomatic infections | 60% | CDC current best estimate (9) |
| Imported cases (daily rate) | Calibrated |  |
| Probability of transmission between one susceptible and one infectious, per contact | Calibrated |  |
| Duration of latent (non-infectious) period, days | 5 | Based on CDC current best estimate (9) |
| Duration of infectious period, days | 2 | Based on CDC current best estimate (9) |
| Natural immunity (median duration, years) | 1 | Assumed, based on literature on human coronaviruses ^17–20^ |
| Relative infectiousness of asymptomatic cases to mild symptomatic | 0.75 | CDC current best estimate (9) |
| Seasonality - month of higher transmission rate | January | Monto et al. (10) |
| Seasonality - maximal impact of the seasonality on transmission rate, % | 0.2 [0-0.4] | Monto et al. (10) |
| Relative severity of recurrent infection (compared to primary infection) | 90% [0-90] | Assumed |

## Model calibration

The model was calibrated using French surveillance data up to November 21, 2020. Three outcomes derived from two different sources were considered: symptomatic cases and death reported by European Centre for Disease Control (ECDC) (11) and hospital admissions reported by Santé Publique France (12).

Data on symptomatic cases were corrected for underreporting using the method proposed by Russel et al. (13). This method assumes that COVID-19 deaths are fully reported and estimates the level of underreporting through a comparison between reported case fatality rate and true case fatality ratio while accounting for the delay between case onset and death. For the delay between case onset and death, we used the distribution reported by Flaxman et al. (14) (gamma distributed with an average of 17.8 days). For the actual case fatality rate, we used the infection fatality ratio estimated by Salje et al. (8) for France to derive an overall case fatality rate of 0.65% in conjunction with the proportion of symptomatic cases defined by the U.S. Centers for Disease Control and Prevention and contact patterns in France.

To account for the evolution over time of the reporting rate of symptomatic cases, the period since start of the pandemic was divided into two subperiods : January-June (includes the first epidemic wave) for which the proportion of reported cases was estimated at 3.4% and July-September during which the proportion of reported cases was estimated at 47%.

We also compared the number of deaths reported by the hospital surveillance system data from Santé Publique France and the number of deaths reported to ECDC for these two periods. For the January-June period, 66% of the deaths reported to ECDC were reported by the hospital surveillance system and 76% for the July-September period. To ensure consistency between these two data sources we used data reported to ECDC as the reference, while adjusting the hospital surveillance system data. When releasing these data, Santé Publique besides mentions that the reporting system is not exhaustive and its level of completeness varies over time (12).

Symptomatic cases corrected for underreporting were used for the calibration of three types of parameters in the model: the contribution of imported cases to the overall transmission parameter ($\beta)$, the contribution of imported cases to the transmission of the disease in the population ($\epsilon_{t})$, and the impact of control measures implemented to prevent transmission ($d_{t})$. The effectiveness of control measures which encompass not only social distancing (school closure, restrictions in public gathering, self-isolation) but also active tracking and isolation of new cases or protection measures such as masks is given by 1-$d_{t}.$ Finally, the contribution of imported cases and impact of control measures are time-dependent and are represented by step functions (step = 5 days). For imported cases, we considered a constant contribution over time from 1 January, 2020 until the first implementation of control measures and nil for the following period. For the impact of control measures, we considered the variation over time using a minimum time period of 5 days. The calibration with least-squares minimization with the nlminb function of the R package stats.

**S2 Fig.** Observed versus simulated evolution of the COVID-19 incidence of symptomatic cases for three outcomes.

**C**

**B**

**A**

**C**

Panel A. Deaths; Panel B. Hospitalisations; and Panel C. Symptomatic cases. Red lines correspond to simulated data for the reference scenario, blue dots represent observed data for the first subperiod (January-June) and grey dots are data for the second subperiod (July-September). The reference scenario: seasonal variation in COVID-19 transmission: 20%, reduced severity of recurrent infection: 90%, medium threshold for non-pharmaceutical interventions initiation.

Finally, we accounted for a specific reduction of contacts post-lockdown of vulnerable people. This reduction in contact pattern could be responsible for the observed shift in the age distribution of hospitalisation. We adjusted the level of exposure to infection of vulnerable people (30% reduction compared to the rest of the rest of the population in the same age) for the second data period. In our simulations, this reduced exposure was maintained until the full discontinuation of NPI measures. The observed and simulated age distribution of hospitalisation is presented in S3 Fig.

**S3 Fig.** Observed (A) and simulated (B) age distribution of COVID-19 hospitalisation by month


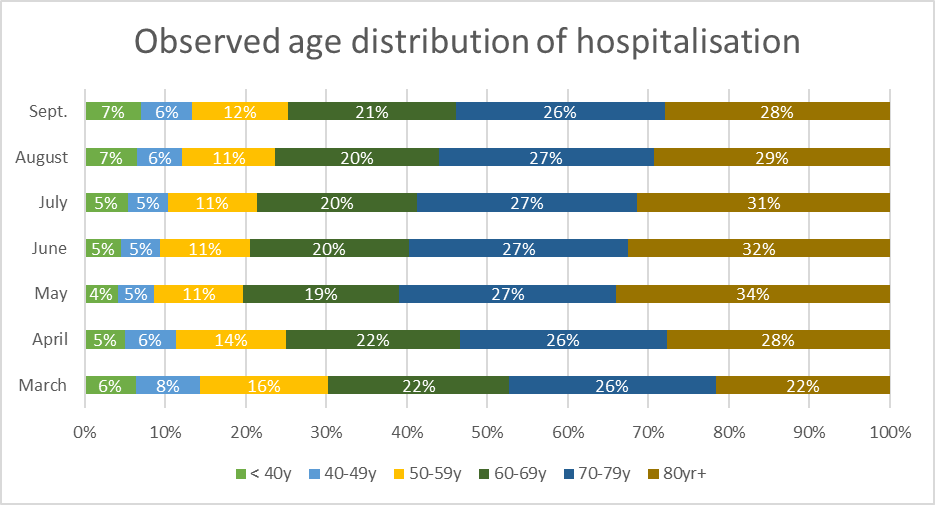

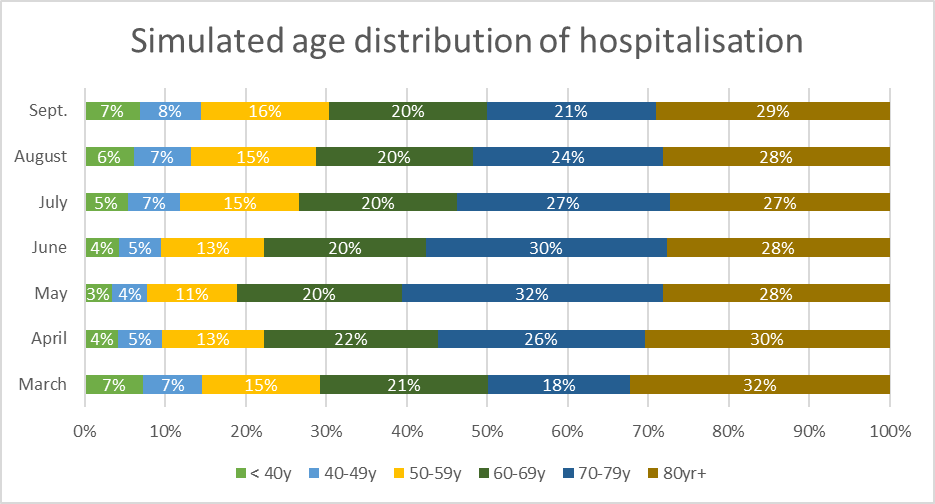


**A**

**B**

## Threshold-based non-pharmaceutical interventions (NPI)

From December 2020, we considered three possible scenarios of threshold-based NPI for mitigating COVID-19 transmission implemented if the level of COVID-19 hospitalisations exceeds a predefined level. Two of these thresholds were based on the peak in incidence observed during the first epidemic wave (April) and the second epidemic wave (November) in France. The third one corresponds to a weak NPI response. We used the second wave threshold as our reference.

We do not explicitly model the effect of specific NPIs but assume a reduction of the effective reproduction number due to a change in measures in place (e.g., social distancing, curfew, lockdown, contract tracing) during NPI activation periods.

Each NPI activation period includes a ramp-up period in order to reach the targeted effective reproduction number. The most stringent measures are maintained until the incidence decreases by 50% compared to the predefined threshold and those measures are progressively relaxed thereafter. The characteristics of the three scenarios considered (first wave threshold, second wave threshold and weak response) are summarized in Table 2.

**S2 Table.** Scenarios of threshold-based NPIs

|  | **First wave threshold** | **Second wave threshold** | **Weak  response** |
| --- | --- | --- | --- |
| Threshold for initiation of stringent measures (incidence of COVID-19 hospitalisations) | 100/1,000,000 | 70/1,000,000 | 200/1,000,000 |
| Targeted effective reproduction number to stop the enforcement of NPIs (Rt)* | 0.9 | 0.9 | 1 |
| Threshold for relaxation of stringent measures (incidence of COVID-19 hospitalisations) | 50/1,000,000 | 37.5/1,000,000 | 100/1,000,000 |
| Timing for full relaxation of stringent measures | 60 days | 60 days | 30 days |

* Daily impact of measures on the level of transmission: 13% reduction in contact rates compared to the previous day i.e. 50% reduction over 5 days.

## Vaccine profiles

We consider two profiles

1. **Protection against infection**

Under this profile, a fraction of vaccinated individuals received full protection against all forms of infection i.e with symptoms or not (defined by $e^{sf},e^{rf}, e^{wf}$ ) but no partial protection ($e^{sp}=e^{rp}=e^{wp}=0$ ) i.e. all-or-nothing vaccine. When vaccine-conferred protection wanes, the risk of infection for previously vaccinated individuals is equivalent to unvaccinated individuals.

In the base case, $e^{sf}=e^{wf}=70\%$ and the median duration of vaccine- conferred protection is set to one year i.e. similar to naturally-acquired protection ($\tau_{v}=\tau_{r}$). Vaccination do not confer any additional protection for subjects protected against infection after a naturally-acquired protection $(e^{rf}=0\%$)

1. **Protection against severity of symptomatic disease**

Under this profile, vaccination has no direct impact on the risk of infection ($e^{sf}=e^{rf}= e^{wf}=e^{i}=0$). Vaccination does not provide additional protection for individuals already infected ($e^{rp}=e^{rw}=$0) but confers partial protection to all susceptible individuals ($e^{sp}=$1).

In the base case, we consider $\frac{p_{i}^{\mathrm{vm}}}{p_{i}^{\mathrm{sm}}}=\frac{p_{i}^{\mathrm{vs}}}{p_{i}^{\mathrm{ss}}}=30\%$ (i.e. 70% reduced severity when compared to infection occurring in susceptible individuals which is lower than the reduced severity for recurrent natural infections in the base case). The vaccine-conferred protection defines the severity of their next infection ($\tau_{v}=100$).

## Vaccine supply

There have been an acceleration and important investment in research and development of COVID-19 vaccines during the first year of the pandemic. While there are over 100 vaccine candidates in clinical development, not all of them will be successful and we will likely see a limited supply compared to the current universal demand during 2021. In Europe, up to December 2020, the European Commission had concluded contracts with six companies covering a broad portfolio of vaccine candidates (see S3 Table). The European Commission has concluded such talks on behalf of its member states.

In this analysis, we assumed that vaccines will be available during the first quarter 2021 in limited supply, and that new supplies will become available mid-year 2021, either due to industrial scale-up of first vaccine(s) or subsequent vaccines becoming available. To calculate the likely supply constraint in January 2021, we mapped the candidates and their expected approval date (S3 Table). Of the six candidates with confirmed supply agreements with the EU, four are expected to become available first quarter of 2021. Supply of vaccines is allocated between member states following a population pro-rata distribution key (15). Taking this into consideration for France, we mapped the purchase commitments (in millions of doses to be provided initially) for these four candidates and the number of doses needed per person. We defined two supply constrained scenarios aiming to cover best- and worst-cases for January 2021. The best-case scenario, a weak supply constraint, assumes enough vaccines become available to vaccinate just under 15 million people by June 2021 and 22 million during the second half of the year. In the worst-case scenario, a strong supply constraint, we assume enough vaccines become available to vaccinate just under six million people by June 2021 and further 12 million people can be then reached by end of the year. These scenarios reflect the range of probable constraints likely to arise in 2021 linked to the advance purchase commitments, implementation and production challenges.

**S3 Table.** EU vaccine commitments for COVID-19 vaccines.

| **Company** | **Platform** | **Doses/ person** | **Expected approval by** | **Millions of doses committed and timing** | | | **Ref** |
| --- | --- | --- | --- | --- | --- | --- | --- |
|  |  |  |  | Jan-21 | Jul-21 | Dec-21 |  |
| AstraZeneca | Non-replicating viral vector | 2 | end 2020 | 300 | 100 |  | (16) |
| Johnson & Johnson | Non-replicating viral vector | 1 | Jan-21 | 200 | 200 |  | (17) |
| Moderna | mRNA | 2 | end 2020 | 80 | 80 |  | (18) |
| BioNtech-Pfizer | mRNA | 2 | Jan-21 | 200 | 100 |  | (19) |
| Sanofi-GSK | Protein-based | 2 | Jul-21 |  |  | 300 | (20) |
| CureVac | mRNA | 2 | end 2021 |  |  | 225 | (21) |

# Detailed results

## Potential evolution of the COVID-19 epidemic in the absence of vaccination and according to level of NPI response

**S4 Fig.** Daily incidence of symptomatic cases (number of cases per million population per day) in the absence of vaccination for varying level of public policy response, 2020-2022.


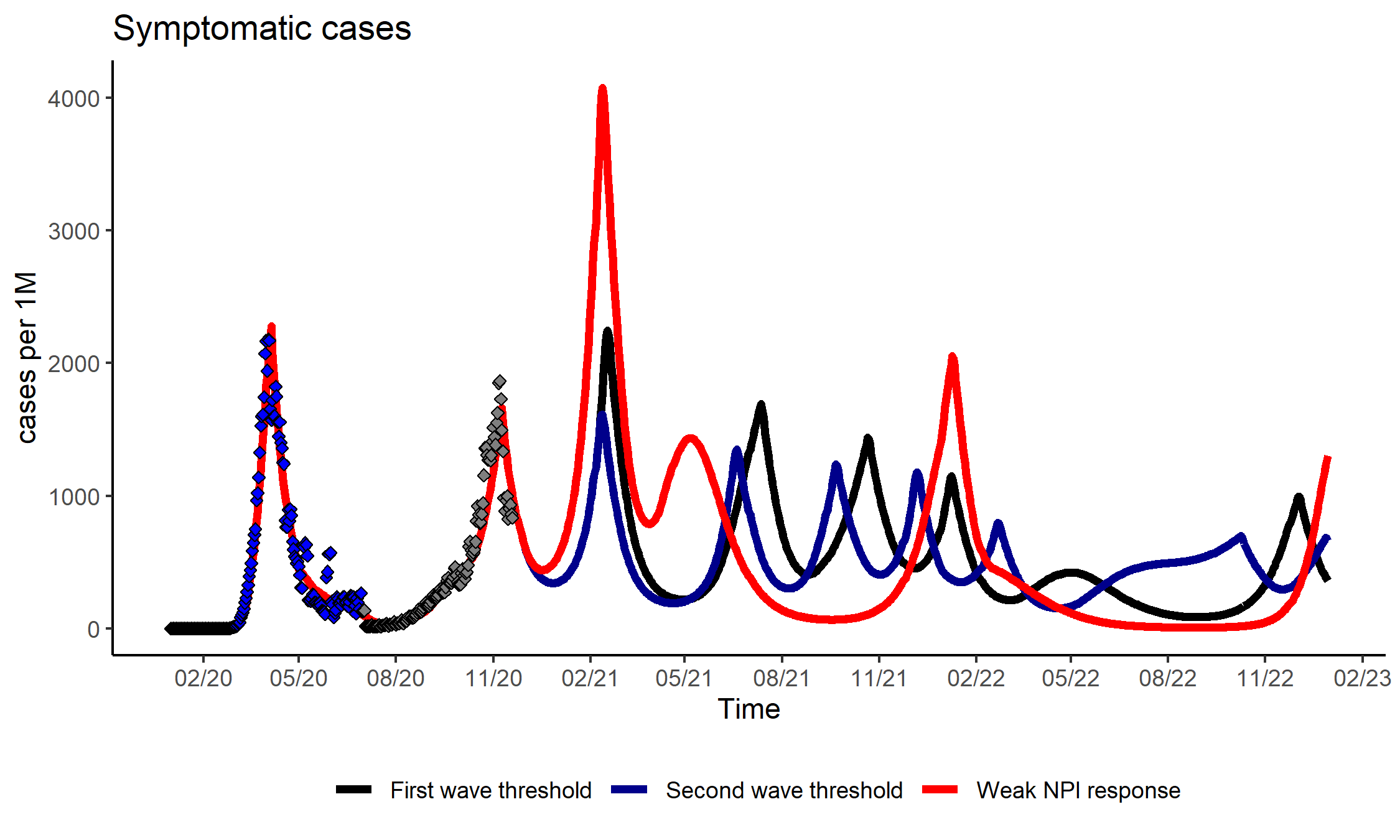


Based on reference scenario for seasonal variation in COVID-19 transmission (20%), severity of reinfection (90% less that primary), median duration of natural/vaccine immunity of one year and NPIs maintained until end of 2022 (second wave threshold).

**S5 Fig.** Daily incidence of COVID-19 deaths (number of deaths per million population per day) in the absence of vaccination for varying level of public policy response, 2020-2022.


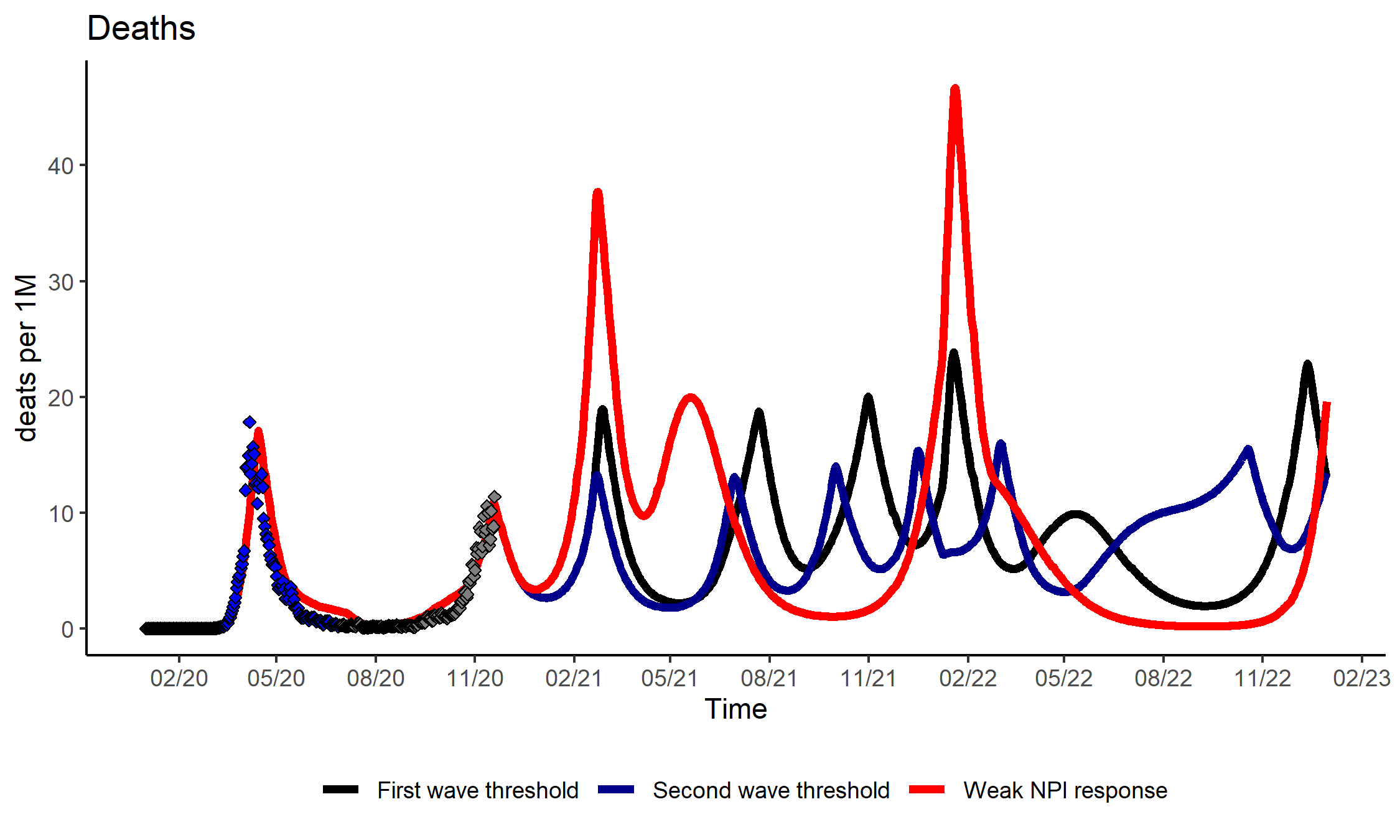


Based on reference scenario for seasonal variation in COVID-19 transmission (20%), severity of reinfection (90% less that primary), median duration of natural/vaccine immunity of one year and NPIs maintained until end of 2022 (second wave threshold).

## Potential mid-term evolution according to timing of NPI discontinuation

**S4 Table.** Cumulative incidence per year of COVID-19 hospitalisation, peak hospitalisation incidence, and number of NPI episodes for varying dates of NPI discontinuation, 2021 and 2022

|  | 2021 | | 2022 | | 2021-2022 | |
| --- | --- | --- | --- | --- | --- | --- |
|  | Incidence | Peak | Incidence | Peak | Incidence | Peak |
| **No vaccination** | | | | | | |
| NPIs stopped 01/01/2021 | 29 754 | 709 | 5 706 | 86 | 35 459 | 709 |
| NPIs stopped 01/01/2022 | 12 769 | 82 | 18 246 | 384 | 31 015 | 384 |
| NPIs stopped 01/07/2022 | 12 769 | 82 | 14 940 | 77 | 27 709 | 82 |
| **Uptake constraint** | | | | | | |
| NPIs stopped 01/01/2021 | 29 043 | 686 | 4 162 | 32 | 33 205 | 686 |
| NPIs stopped 31/12/2021 | 10 959 | 79 | 6 291 | 124 | 17 250 | 124 |
| NPIs stopped 31/12/2022 | 10 960 | 79 | 5 666 | 76 | 16 626 | 79 |
| **Strong supply constraint** | | | | | | |
| NPIs stopped 01/01/2021 | 29 089 | 686 | 4 385 | 53 | 33 474 | 686 |
| NPIs stopped 31/12/2021 | 12 817 | 79 | 10 369 | 179 | 23 186 | 179 |
| NPIs stopped 31/12/2022 | 12 819 | 79 | 9 837 | 75 | 22 656 | 79 |
| **Weak supply constraint** | | | | | | |
| NPIs stopped 01/01/2021 | 29 050 | 686 | 4 154 | 41 | 33 204 | 686 |
| NPIs stopped 31/12/2021 | 12 572 | 79 | 5 397 | 96 | 17 969 | 96 |
| NPIs stopped 31/12/2022 | 12 573 | 79 | 5 359 | 80 | 17 932 | 80 |
| **Relaxed strong supply and uptake constraints** | | | | | | |
| NPIs stopped 01/01/2021 | 29 088 | 686 | 2 333 | 20 | 31 421 | 686 |
| NPIs stopped 31/12/2021 | 11 629 | 79 | 3 185 | 78 | 14 814 | 79 |
| NPIs stopped 31/12/2022 | 11 630 | 79 | 3 181 | 74 | 14 811 | 79 |
| **Relaxed weak supply and uptake constraints** | | | | | | |
| NPIs stopped 01/01/2021 | 29 050 | 686 | 2 075 | 37 | 31 124 | 686 |
| NPIs stopped 31/12/2021 | 9 240 | 79 | 1 699 | 66 | 10 939 | 79 |
| NPIs stopped 31/12/2022 | 9 240 | 79 | 1 702 | 66 | 10 942 | 79 |

Incidence: hospitalisations per million population per year; Peak: Peak hospitalisation incidence during a particular year; Reference scenario for disease characteristics, NPI response (second wave threshold) and vaccine profile (70% efficacy against infection).

## Potential vaccination impact

**S5 Table.** Cumulative incidence of symptomatic cases per million population per period and percentage reduction for immunisation programs with and without relaxation of constraints compared to no vaccination, 2021-2022.

|  | 2021 | | 2022 | | 2021-2022 | |
| --- | --- | --- | --- | --- | --- | --- |
|  | Incidence | % | Incidence | % | Incidence | % |
| No vaccine | 218 970 | ref | 152,192 | ref | 371,162 | ref |
| **Immunization program under constraints** | | | | | | |
| Strong supply constraint | 225,250 | 2.9% | 109,888 | -27.8% | 331,762 | -10.6% |
|  | [212587;228021] | [-3;4] | [103736;116115] | [-32;-24] | [331762;329888] | [-11;-9] |
| Weak supply constraint | 225,266 | 2.9% | 70,993 | -53.4% | 307,075 | -17.3% |
|  | [219183;244290] | [0;12] | [62785;102144] | [-59;-33] | [293132;310968] | [-21;-13] |
| Uptake constraint | 217,040 | -0.9% | 64,503 | -57.6% | 29,0148 | -21.8% |
|  | [207236;233565] | [-5;7] | [57940;81614] | [-62;-46] | [271355;291506] | [-27;-20] |
| **Immunization program with relaxed constraints** | | | | | | |
| Relaxed strong supply and uptake constraint | 216,725 | -1.0% | 6,2345 | -59.0% | 275,256 | -25.8% |
|  | [209236;233654] | [-4;7] | [39344;70276] | [-74;-54] | [267690;275256] | [-28;-25] |
| Relaxed weak supply and uptake constraint | 202,958 | -7.3% | 36,135 | -76.3% | 238,851 | -35.6% |
|  | [196115;222260] | [-10;2] | [29631;45585] | [-81;-70] | [238851;235310] | [-37;-32] |

Based on reference scenario for disease characteristics, NPI response (second wave threshold), vaccine profile (70% efficacy against infections) and NPIs maintained until end of 2022. Incidence is given per million population and the % of variation is calculated in reference to the no vaccine counterfactual. For each scenario, both median values and range are provided for a vaccine efficacy ranging from 50% to 90%.

**S6 Table.** Cumulative incidence of COVID-19 deaths per million population per period and percentage reduction for immunisation programs with and without relaxation of constraints compared to no vaccination, 2021-2022.

|  | 2021 | | 2022 | | 2021-2022 | |
| --- | --- | --- | --- | --- | --- | --- |
|  | Incidence | % | Incidence | % | Incidence | % |
| No vaccine | 2,254 | ref | 3,151 | ref | 5,405 | ref |
| **Immunization program under constraints** | | | | | | |
| Strong supply constraint | 2,220 | -1.5% | 2,018 | -36.0% | 4,234 | -21.7% |
|  | [2059;2265] | [-9;0] | [1881;2204] | [-40;-30] | [4146;4234] | [-23;-20] |
| Weak supply constraint | 1,943 | -13.8% | 1,069 | -66.1% | 3,163 | -41.5% |
|  | [1866;2094] | [-17;-7] | [987;1813] | [-69;-42] | [2853;3461] | [-47;-30] |
| Uptake constraint | 1,849 | -18.0% | 1,091 | -65.4% | 2,974 | -45.0% |
|  | [1638;2000] | [-27;-11] | [926;1486] | [-71;-53] | [2564;3091] | [-53;-37] |
| **Immunization program with relaxed constraints** | | | | | | |
| Relaxed strong supply and uptake constraint | 2,061 | -8.6% | 462 | -85.3% | 2,523 | -53.3% |
|  | [1968;2198] | [-13;-2] | [242;811] | [-92;-74] | [2370;2595] | [-56;-49] |
| Relaxed weak supply and uptake constraint | 1,627 | -27.8% | 259 | -91.8% | 1,928 | -64.3% |
|  | [1560;1919] | [-31;-15] | [224;323] | [-93;-90] | [1824;1935] | [-67;-59] |

Based on reference scenario for disease characteristics, NPI response (second wave threshold), vaccine profile (70% efficacy against infections) and NPIs maintained until end of 2022. Incidence is given per million population and the % of variation is calculated in reference to the no vaccine counterfactual. For each scenario, both median values and range are provided for a vaccine efficacy ranging from 50% to 90%.

**S7 Table.** Number of symptomatic cases averted with and without constraint relaxation, 2021-2022.

|  | **Without relaxation in July^1^** | | **With relaxation in July^2^** | |
| --- | --- | --- | --- | --- |
|  | Median | Range | Median | Range |
| **Strong supply constraint** | | | | |
| Cases averted | 39,400 | [33724;42460] | 58,369 | [49191;64072] |
| Vaccinated subjects | 181,719 | [181677;181739] | 341,464 | [341427;341490] |
| Cases averted/1,000 vaccinated subjects | 216.9 | [185.6;233.6] | 170.9 | [144;187.7] |
| Variation in incidence (%) | -10.6% | [-11;-9] | -17.3% | [-19;-15] |
| Days with NPIs | 393 | [387;498] | 269 | [207;285] |
| **Weak supply constraint** | | | | |
| Cases averted | 64,087 | [48458;78030] | 69,524 | [54214;75658] |
| Vaccinated subjects | 340,574 | [340524;340578] | 341,535 | [341512;341564] |
| Cases averted/1,000 vaccinated subjects | 188.2 | [142.3;229.1] | 203.6 | [158.7;221.5] |
| Variation in incidence (%) | -17.3% | [-21;-13] | -21.5% | [-24;-18] |
| Days with NPIs | 208 | [149;377] | 203 | [149;212] |

Based on reference scenario for disease characteristics, NPI response (second wave threshold), vaccine profile (protection against infection) and NPIs maintained until end of 2022. 1: Compared to the no vaccine counterfactual. 2: Compared to the corresponding constrained scenario. Cases averted: Cases averted are per million population; Vaccinated subjects: people vaccinated per million population; Cases averted/1,000 vaccinated subjects: Cases averted per 1,000 vaccinated people. For each scenario, both median values and range are provided for a vaccine efficacy ranging from 50% to 90%.

**S8 Table.** Number of COVID-19 deaths averted with and without constraint relaxation, 2021-2022.

|  | **Without relaxation in July^1^** | | **With relaxation in July^2^** | |
| --- | --- | --- | --- | --- |
|  | Median | Range | Median | Range |
| **Strong supply constraint** | | | | |
| Deaths averted | 1,171 | [1080;1259] | 1,726 | [1485;1802] |
| Vaccinated subjects | 181,719 | [181677;181739] | 341,464 | [341427;341490] |
| Deaths averted/1,000 vaccinated subjects | 6.4 | [5.9;6.9] | 5.1 | [4.3;5.3] |
| Variation in incidence (%) | -21.7% | [-23;-20] | -41.2% | [-43;-35] |
| Days with NPIs | 393 | [387;498] | 269 | [207;285] |
| **Weak supply constraint** | | | | |
| Deaths averted | 2,242 | [1632;2552] | 1,362 | [1029;1531] |
| Vaccinated subjects | 340,574 | [340524;340578] | 341,535 | [341512;341564] |
| Deaths/1,000 vaccinated subjects | 6.6 | [4.8;7.5] | 4.0 | [3;4.5] |
| Variation in incidence (%) | -41.5% | [-47;-30] | -40.6% | [-44;-36] |
| Days with NPIs | 208 | [149;377] | 203 | [149;212] |

Based on reference scenario for disease characteristics, NPI response (second wave threshold), vaccine profile (protection against infection) and NPIs maintained until end of 2022. 1: Compared to the no vaccine counterfactual. 2: Compared to the corresponding constrained scenario. Deaths averted: Deaths averted are per million population; Vaccinated subjects: people vaccinated per million population; Deaths averted/1,000 vaccinated subjects: Deaths averted per 1,000 vaccinated people. For each scenario, both median values and range are provided for a vaccine efficacy ranging from 50% to 90%.

**S6 Fig.** Tornado diagram of number of NPI days in 2022 for different vaccine and disease characteristics (relaxed strong supply and uptake constraints scenario.).


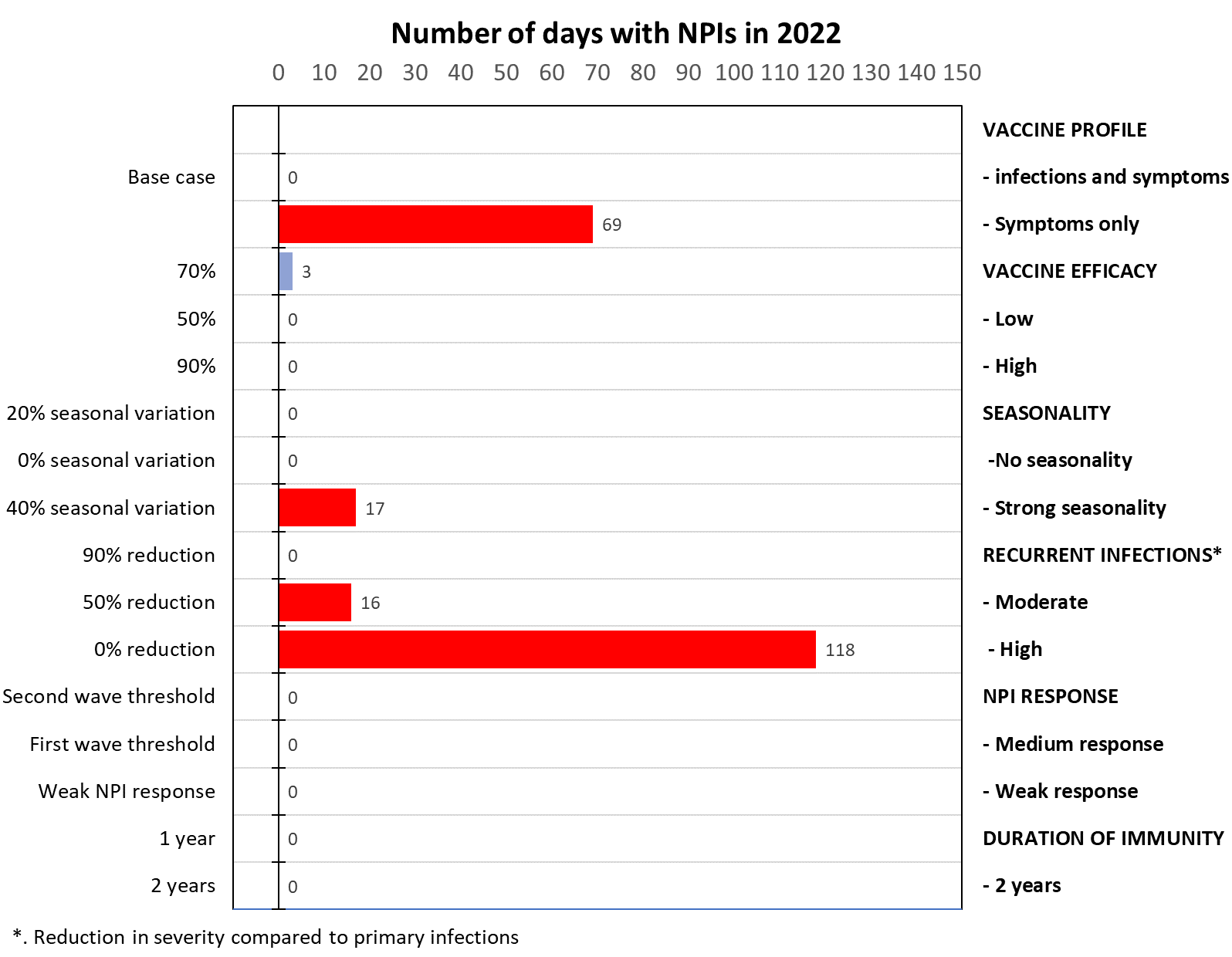


All outcomes presented corresponds to univariate sensitivity analysis of the reference case for key disease and vaccine characteristics. The figure indicates the number of days with implementation of NPIs in 2022.

**S7 Fig.** Tornado diagram of reduction in hospitalisations and peak incidence of hospitalisations, 2021-2022 (Uptake constraint scenario).


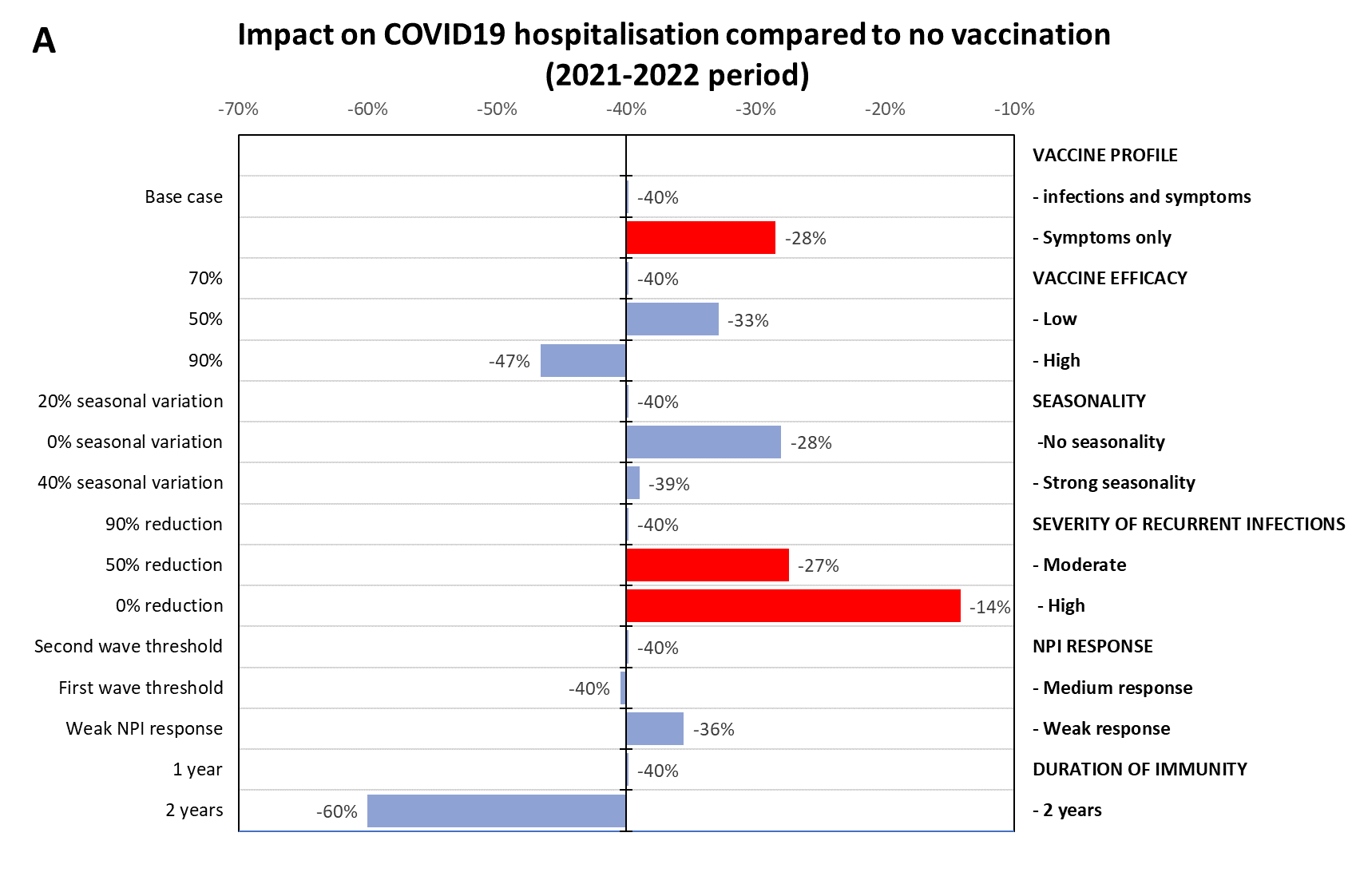


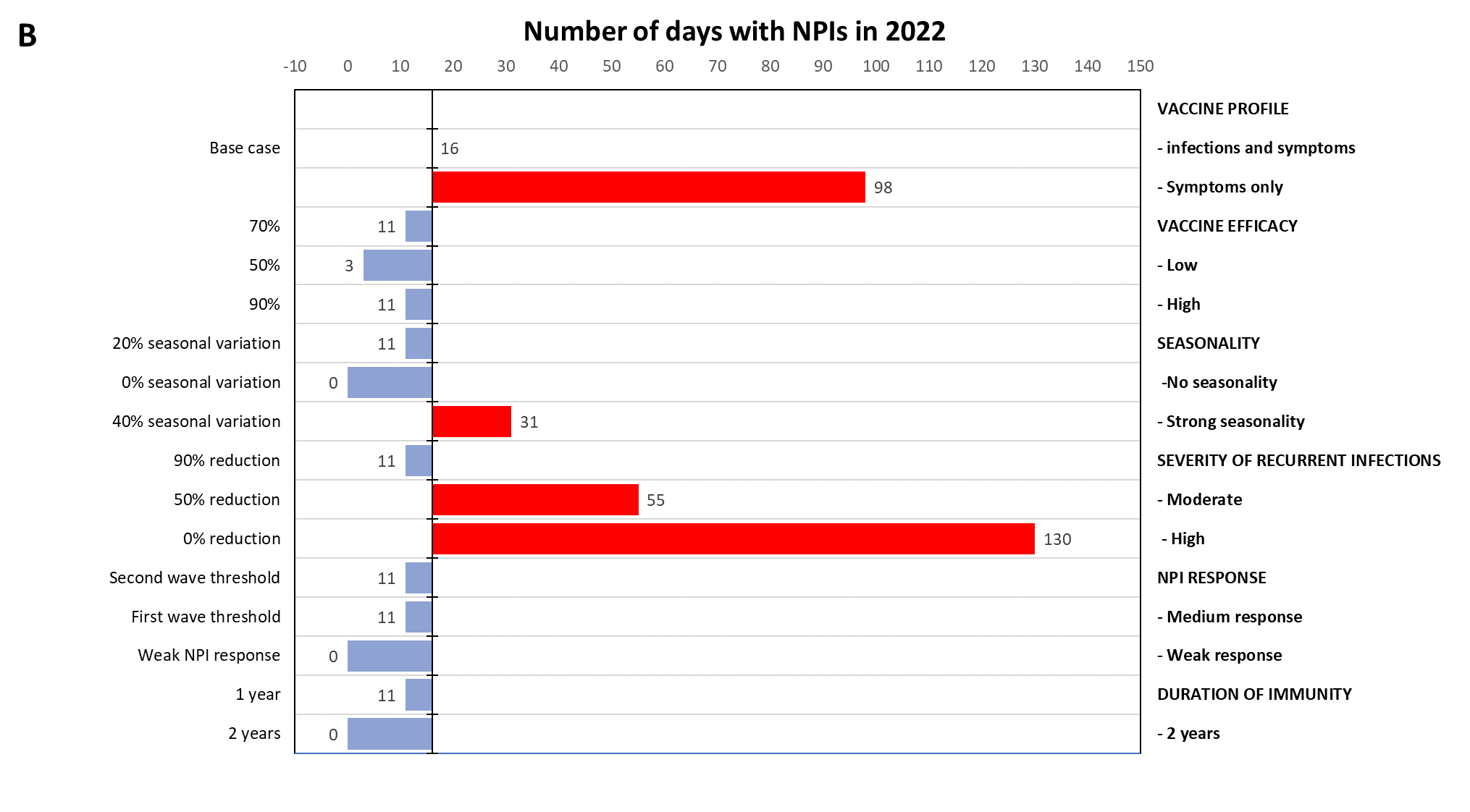


All outcomes presented corresponds to univariate sensitivity analysis of the reference case for key disease and vaccine characteristics. **Panel A** shows the change in number of hospitalisations (as %) for the 2021-2022 period compared to no vaccination counterfactual. **Panel B** indicates the number of days with implementation of NPIs in 2022.

**References**

1. Coudeville L, Gomez G, Jollivet O, Harris R, Thommes E, Druelles S, et al. Exploring uncertainty and risk in the accelerated response to a COVID-19 vaccine: Perspective from the pharmaceutical industry. Vaccine. 2020.

2. Hethcote HW. The Mathematics of Infectious Diseases. SIAM Review. 2000;42(4):499-633.

3. Team RC. R: A language and environment for statistical computing. 2013.

4. Team R. RStudio: integrated development for R. 2015. RStudio, Inc, Boston, MA URL <http://www> rstudio com Accessed. 2017;7.

5. Soetaert KE, Petzoldt T, Setzer RW. Solving differential equations in R: package deSolve. Journal of Statistical Software. 2010;33.

6. United Nations (UN) DoEaSA, Population Division. World Population Prospects 2019 [Available from: <https://population.un.org/wpp/>).

7. Prem K, Cook AR, Jit M. Projecting social contact matrices in 152 countries using contact surveys and demographic data. PLoS Comput Biol. 2017;13(9):e1005697.

8. Salje H, Tran Kiem C, Lefrancq N, Courtejoie N, Bosetti P, Paireau J, et al. Estimating the burden of SARS-CoV-2 in France. Science. 2020;369(6500):208-11.

9. CDC. COVID-19 Pandemic Planning Scenarios 2020 [Available from: <https://www.cdc.gov/coronavirus/2019-ncov/hcp/planning-scenarios.html>.

10. Monto AS, DeJonge PM, Callear AP, Bazzi LA, Capriola SB, Malosh RE, et al. Coronavirus Occurrence and Transmission Over 8 Years in the HIVE Cohort of Households in Michigan. J Infect Dis. 2020;222(1):9-16.

11. ECDC. Geographic distribution of COVID-19 cases worldwide 2020 [Available from: <https://www.ecdc.europa.eu/en/publications-data/download-todays-data-geographic-distribution-covid-19-cases-worldwide>.

12. France SP. Données hospitaliéres relatives à l'épidémie de COVID-19 2020 [Available from: <https://www.data.gouv.fr/fr/datasets/donnees-hospitalieres-relatives-a-lepidemie-de-covid-19/>.

13. Russell TW, Hellewell J, Jarvis CI, Van Zandvoort K, Abbott S, Ratnayake R, et al. Estimating the infection and case fatality ratio for coronavirus disease (COVID-19) using age-adjusted data from the outbreak on the Diamond Princess cruise ship, February 2020. Eurosurveillance. 2020;25(12):2000256.

14. Flaxman S, Mishra S, Gandy A, Unwin HJT, Mellan TA, Coupland H, et al. Estimating the effects of non-pharmaceutical interventions on COVID-19 in Europe. Nature. 2020;584(7820):257-61.

15. Commission E. Preparedness for COVID-19 vaccination strategies and vaccine deployment 2020 [Available from: <https://ec.europa.eu/health/sites/health/files/vaccination/docs/2020_strategies_deployment_en.pdf>.

16. Commission E. Coronavirus: the Commission signs first contract with AstraZeneca Brussels: European Commission: Press corner; 27/10/2020 [Available from: <https://ec.europa.eu/commission/presscorner/detail/en/IP_20_1524>.

17. Commission E. Coronavirus: Commission concludes further talks to secure future vaccine: European Commission: Press corner; 13/08/2020 [Available from: <https://ec.europa.eu/commission/presscorner/detail/en/ip_20_1481>.

18. Commission E. Coronavirus: Commission expands talks to a fifth vaccine manufacturer Brussels: European Commission: Press corner; 24/08/2020 [Available from: <https://ec.europa.eu/commission/presscorner/detail/en/IP_20_1513>.

19. Commission E. Coronavirus: Commission completes vaccines portfolio following talks with a sixth manufacturer Brussels: European Commission: Press corner; 09/09/2020 [Available from: <https://ec.europa.eu/commission/presscorner/detail/en/ip_20_1556>.

20. Commission E. Coronavirus: Commission concludes talks to secure future coronavirus vaccine for Europeans Brussels: European Commission: Press corner; 31/07/2020 [Available from: <https://ec.europa.eu/commission/presscorner/detail/en/ip_20_1439>.

21. Commission E. Coronavirus: Commission continues expanding future vaccines portfolio with new talks Brussels: European Commission: Press corner; 20/08/2020 [Available from: <https://ec.europa.eu/commission/presscorner/detail/en/IP_20_1494>.
