# Supplementary material for: Potential impact of introducing vaccines against COVID-19 under supply and uptake constraints in France: A modelling study
Source: PLoS One. 2021 Apr 28;16(4):e0250797. doi: 10.1371/journal.pone.0250797 (PMC8081204; doi:10.1371/journal.pone.0250797)
Supplement: Attachment — Submitted filename: Response to Reviewers.docx [file pone.0250797.s003.docx]

# Response to reviewers

We would like to thank the editor and reviewers for their thorough review of our manuscript and insightful comments. In this revised version, we aimed at addressing each of the comments as indicated in our point-by-point response below.

**Reviewer #1: The study is a statistic model of the vaccine effect on public health. the study adds to previous literature analysis on timing of benefits of future immunization program. The study is published when there are already preliminary data on the efficacy of the vaccine and therefore it is published a little too late (After the horses are already out of the barn). there is still benefit in publishing as the model predicts the morbidity in the next two years.**

🡪 We agree with the reviewer that other modelling studies have addressed the issue of impact of future immunization programs. These studies have explored the parameter space of vaccine efficacy. As the reviewer points out, efficacy trials have been published and we are now in the process of expanding vaccination coverage. An analysis placing the constraints (supply and uptake) we are having in program expansion is timely and needed to inform the current discourse. Currently, demand is outstripping supply, governments and health systems are facing challenges in implementation of what is arguably the biggest immunization program in history. In addition, uptake constraints (for example a reluctance to be vaccinated) could potentially pose serious challenges to programs in countries such as France. Therefore, our message can reinforce the need of programs to tackle these issues sooner rather than later. We tried to reinforce this message (of timeliness) for the benefit of readers in the first paragraph of the discussion.

**The study claim that the vaccine will need to be highly effective and to achieve high coverage to be able to obviate the need of non-pharmaceutical interventions and to control the pandemic. The data support the claim but there are some major comments:**

**Introduction:
1. Line 4- the numbers should be updated (for example there are more than 20 ongoing phase III trials and not 11).**

🡪 we updated these numbers – it is indeed a rapidly evolving field. If accepted, we will also ensure these number are up to date in the proofs.

**2. Line 8- the number of vaccine candidates should be updated (there are at least 3 authorized vaccines already).**

**🡪** we updated these numbers. If accepted, we will also ensure these number are up to date in the proofs.

**3. Line 23- please add reference to the study that was mentioned (modelling by the imperial college…).**

🡪 Apologies to the reviewers and editor – we experienced a glitch when generating the reference list and the numbers do not correspond to the references meant. We have verified all references.

**4. Line 23- references 13 and 14 are actually a survey results about vaccine hesitancy and less about strategies to optimize immunization. Please add relevant reference.**

🡪 same as above

**5. Line 25- reference 15 is a study about the willingness of the population to be vaccinated. I couldn't find any support in the authors claim about supply constraint. Another reference would be more suitable.**

🡪 same as above

**Methods:
1. All the references should be checked. For example hospital admission reported by sante was specified as Ref 24 when it is actually Ref 25.**

🡪 same as above

**2. The definitions of uptake constraints and supply constraints are confusing and not intuitive. For example the phrase 'Relaxed uptake constraints' is confusing and not intuitively understood as high compliance. I suggest change 'uptake constraints' to more simple definition like 'vaccine compliance', and supply constrains to vaccine supply or quantity.**

🡪 we have five scenarios of varying constraints. The terminology aims to reflect the programmatic evaluation literature and we want to highlight the limitation of programs as well as the dynamic aspect of the limitations. To clarify, we have added the following explanations to table 1.

- Uptake constraint reflects a limited coverage rate due to a limited willingness to be vaccinated in the population.
- Strong supply constraint reflects a limited amount of vaccines doses made available to the national program. In this case, the program is severely limited.
- Weak supply constraint reflects a limited amount of vaccines doses made available to the national program, in this case the program is moderately limited.
- Relaxed strong supply and uptake constraints. In this scenario, while the program is severely limited during the first half of the year, vaccine supply is increased during the second half of the year (higher production or new vaccines availability) and the public is more likely to be willing to vaccinate as the program has been in place for half a year. Therefore, we ‘eased’ the limitations of the program in the second semester.
- Relaxed weak supply and uptake constraints. In this scenario, while the program is moderately limited during the first half of the year, vaccine supply is increased during the second half of the year and the public is even more likely to be willing to vaccinate as the program has been in place for half a year. Therefore, we ‘eased’ the limitations of the program to achieve maximum coverage.

**3. Line 43- please mention what period was taken.**

🡪 we have added the duration of such immunity period – Median duration of one year.

**4. Line 79- the association of other vaccines, such as influenza vaccine, have been proved to reduce COVID-19 infection(doi: 10.1080/21645515.2020.1852010). It can therefore be assumed that a dedicated vaccine will prevent disease and not only reduce symptoms. I suggest discuss it and take it into account when assuming vaccine efficacy.**

🡪 We agree with the reviewer that given experience gathered on other vaccines, the ability for vaccines to impact COVID-19 infections and hence reduce transmission is a likely scenario. We besides considered for our reference case a vaccine that prevents infection. This has however not been fully demonstrated to date even if some preliminary evidence exists (<https://papers.ssrn.com/sol3/papers.cfm?abstract_id=3777268>, <https://doi.org/10.1101/2021.02.08.21251329>)

Regarding the link between COVID-19 and influenza vaccination, even if the publication mentioned by the reviewer indicates positive impact of previous influenza vaccination, other studies have not found this association (<https://academic.oup.com/occmed/article/70/9/665/6029444> and <https://onlinelibrary.wiley.com/doi/10.1111/irv.12839>).

In this revised version, we indicate that a vaccine preventing infection is the most likely scenario and acknowledged remaining uncertainties. We however did not discuss the link between COVID-19 vaccination and previous influenza vaccination we considered is beyond the scope of our analysis.

**5. Line 83- the authors mentioned efficacy of 50-90%. Previous studies mentioned 95% efficacy of the vaccine (for example: DOI: 10.1056/NEJMoa2034577). Such a large variation in the efficacy data of the vaccine may alter the results of the statistical model.**

🡪 We have rephrased the paragraph on vaccine’s profiles modelled to reflect current knowledge available for already approved vaccines. The results are presented for a vaccine efficacy of 70% with shaded areas representing a variation from 50-90%. While efficacy in clinical trials with short follow up has been shown to be 95% for some of the vaccines, we wanted to provide a range that represents the mix of vaccine effectiveness (real life estimates) likely to be available, representing scenarios and not necessarily guessing actual estimates. Moreover, we are still uncovering results of vaccine effectiveness on reduction of infection (i.e., asymptomatic and symptomatic infections). We have added text to the figures, so that ranges of efficacy are clearly represented.

**6. Line 99- the vaccine uptake was set as 60%. On what was the assessment based? There are published surveys on compliance that the author can rely on (for example: DOI: 10.1016/j.vaccine.2020.08.043).**

🡪 We based the 60% uptake on published surveys of willingness to vaccinate attitudes similar to the one suggested by the reviewer but specific to France. We have reworded the text to reflect the inclusion of this evidence.

**7. Line 109 -the details are not accurate. There are already a few available vaccines. Assumption is not needed.**

🡪 we have reviewed the statements on vaccine availability in Europe to include further exploratory talks started mid-January and the authorizations to date. Our scenarios reflect supply constraints that are not only related to the authorization of vaccines but also the industrial capacity of companies to deliver at the scale needed. In fact, despite initial projections of vaccine dose availability, most countries in Europe and in North America are facing supply constraints due to production limitations (similar to the constraint scenarios assumptions). We added text to the methods to back up our assumptions.

**8. Line 130- the sentence is to complicate and long. Please simplify.**

🡪 We have simplified the text to clarify what is present in the main text as opposed to the additional results.

**Results:
1. To my point of view, mean and C.I instead of median and range will be more appropriate.**

🡪 we present the values for our reference case, a vaccine efficacy of 70% and the range represents the variation in results of our range of efficacy considered. We have clarified this in each figure., table and the results.

**2. Line 184- reduction in hospitalization range is not presented in table 3. Please clarify.**

🡪 The range mentioned line 184 (30 to 39) corresponds respectively to the lower and upper values of the confidence intervals corresponding to the two vaccination scenarios with constraint we considered in our manuscript. To clarify this point, we modified the sentence to indicate explicitly the two confidence intervals reported Table 3.

**3. The effect of the vaccine compliance and supply was measured as hospitalization incidence. I think that number of infected patients is more appropriate. Please explain why did you choose that variable.**

🡪 we chose to present hospitalizations in the main text as it is a locally relevant indicator of the stress COVID-19 is putting on the health system which leads to government decisions on implementing mitigation measures. However, we acknowledge this is not the only relevant indicator. We present additional indicators such as number of days with NPIs in place in the main text, while cases and deaths are presented in the appendix. We have clarified this in the methods section and discussion.

**4. Previous study has demonstrated that hemoglobin A1C is a predictor of COVID‐19 Severity (doi: 10.1002/dmrr.3398 ). Has a connection between adherence to diabetes treatment and infectivity (R) and response to the vaccine been taken into account?**

🡪 There may be an association between hemoglobin A1C and COVID-19 severity as pointed out by the reviewer and which is still explored by some researchers. However, the relevance of such predictor in the context of our analysis is accounted for by using broader risk categories driven by local literature on fatality ratio, hospitalization rates and social contact matrices. Moreover, our analysis did not aim to account for the effect of every individual risk factor, varying individual immune response and all the angles of constraints associated with rollout of vaccination programs for COVID-19. Our manuscript demonstrates scenarios driven by uncertainties and discuss the importance of considering such uncertainties when planning public health interventions.

**5. Currently, there is a growing evidence of side effects of the vaccine. Was it taken into account in calculating hospitalization rates and compliance rates?**

🡪 Currently, the hospitalization rates and uptake are based on the reported rates for France and the willingness to vaccinate on surveys, respectively. As both pieces of evidence are pre-vaccine, the hospitalization rates or uptake rates do not account for changes since the start of the program. However, the program started 3 weeks ago and there is no data available. We added this as a limitation to the discussion.

**Tables:
1. Table 2- please rechecked the numbers (hospitalization rates are higher under 'vaccine strong constraints' than under 'no vaccine' at all).**

🡪 The result for the strong supply constraint scenario indeed indicates a potential slight increase in hospitalization in 2021 (range: -6 to +2%) but not for the 2021-2022 period (range : -18 to -21%) and not for all other scenarios. This rather counterintuitive result stems from the fact that the vaccination program indirectly impacts the level of NPI in our analysis which remains an important driver for the overall COVID-19 burden in 2021 (lower NPIs are associated with a larger number of hospitalizations). As it corresponds to a rather extreme scenario and is no longer observed when a more adequate time horizon for assessing vaccination benefits is considered (i.e. 2021-2022), this result should not be overstated. Nevertheless, it reinforces the message in our manuscript around the need for a combination of vaccination and NPI in 2021.

**Reviewer #2: In this manuscript the authors present an analysis of the COVID-19 spreading dynamics in France after the initiation of vaccination drive. The incorporation of constraints from both the supply and demand side adds realism to the model. Some statements from the results section drew my attention however. They are : (a) that even with the uptake constraint i.e. maximal vaccine distribution, there would be NPIs being applied in 2022,**

**(b) that with discontinuation of NPI at the end of 2021, there would be +0·8 percent, -9·4 percent and (presumably negative although this has not been indicated by authors) 13·9 percent variation in hospitalizations in 2021 with the three vaccine constraints relative to the no vaccine case,**

**and (c) that the incidence of COVID-19 remains significant at the end of 2022 with all situations of vaccination and even on the rise for most of them.**

**These statements are counter-intuitive and a bit pessimistic – most people are hoping for a return to normalcy by this fall or at least by the end of the year. I would like the authors to recognize and discuss this fact in detail.**

🡪 In this analysis, we focused in the short-term impact of a vaccination program taking place during 2021 with a specific focus on the role of supply and uptake constraints. Even if some our conclusions might appear pessimistic to the reviewer, our conclusions around the need to maintain NPIs alongside vaccination are actually consistent with several other recent pre-publications see e.g. <https://doi.org/10.1101/2021.01.06.21249339> , <https://doi.org/10.1101/2020.12.30.20248888> . In any case, our analysis can contribute to the debate around the need to tackle issues related to programmatic issues associated to vaccination programs

With regards to COVID-19 activity observed post 2021, our results notably reflect assumptions on duration of immunity and the fact that we did not specifically consider the implementation of routine vaccination in 2022 as it is beyond the scope of the analysis presented in our manuscript.

We however acknowledge that there are clearly remaining issues with regards to the future evolution of COVID-19. In this revised version, we expanded the discussions notably the limitations of the study to hopefully clarify our conclusions and put them in regards with the current knowledge on COVID-19.

We also thank the reviewer for identifying the missing minus sign before 13.9, this was corrected in main text (already accurate in table 2).

**The authors could consider several major and minor revisions. Some major revisions are given below:**

**1 There are certain modeling studies of vaccination dynamics which present a more optimistic view than the authors’ work. A key example is
Shayak B, Sharma MM and Mishra AK, “Impact of immediate and preferential relaxation of social and travel restrictions for vaccinated people on the spreading dynamics of COVID-19 : a model-based analysis,” available at**
[**https://www.medrxiv.org/content/10.1101/2021.01.19.21250100v1**](https://www.medrxiv.org/content/10.1101/2021.01.19.21250100v1) **The references by Alvarez et. al. and Betti et. al. in the above manuscript are also optimistic. While I am aware that all these works were written after the authors’ manuscript, they must now be cited. It must be explained why the authors’ results differ from these analyses.**

🡪 thank you for pointing these papers out. We have referenced and added a commentary in the discussion to place our results in the context of these other studies.

**2 What is the role of the temporary immunity in generating the authors’ case trajectories ? In other words, if the vaccine immunity had been 2 years or 5 years, then what would the trajectories have been like ? Authors should perform simulations to demonstrate this.**

🡪 We made the choice in this manuscript to focus on programmatic constraints without necessarily exploring in detail the duration of immunity, especially as our period of analysis is relatively short (2 years including one year of vaccination). This is the topic of other papers in the field (see e.g. <https://doi.org/10.1126/science.abd7343>) and in a context where the short-term evolution of COVID-19 remains mainly driven by the primary infections and level of NPIs this does not necessarily significantly impact our conclusions even if a longer duration of immunity improved expected vaccination benefits.

Below, we illustrate the evolution in the absence of vaccination for a duration of immunity varying from 1 to 5 years. For a 2 years duration of naturally immunity, it is still expected to have a significant COVID-19 circulation in 2022.

In the revised version, we updated Figure 4 to include a scenario with a media duration of immunity of 2 years and also added discussion on this point.

**3 What is the role of the bang-bang NPI control strategy in generating the authors’ bleak predictions ? Instead of this strategy, if a continuous NPI were applied or NPI gradually relaxed over time then what would the trajectories have looked like ? Simulations should be performed to analyse these questions.**

🡪 We considered in our analysis two types of NPIs: threshold-based NPIs i.e. more stringent measures implemented when incidence exceeds a predefined threshold but also risk-based NPIs i.e. reduced exposure to infection for vulnerable people compared to the rest of the population. Contrary to the first one, this second one is continuous throughout the roll-out of the vaccination program.

The threshold-based NPIs, that can be seen as a stop-and-go approach however account for a gradual relaxation of measures over time. Time-varying measures are also in fact consistent with actual measures implemented in a number of countries including France. Fully constant NPIs will not necessarily represent reality (even if authorities do not enforce any change in their policy a more active virus circulation can impact individual behavior). Besides, one of the main expected outcomes of vaccination is the ability to stop NPIs and the impact of vaccination on NPIs is part of the analysis we performed.

We however agree with the reviewer that more constant NPIs could have been considered in our analysis (e.g. longer period for relaxation of NPIs after an outbreak) but this won’t have changed our main conclusion. In this revised, we explicitly mention as a limitation that we did not account for all possible evolutions of NPIs over time.

**4 Why there is an increase in hospitalization in 2021 with strong supply constraint relative to no vaccination ? Surely this is a surprising result.**

🡪 (similar response to reviewer 1): The result for the strong supply constraint scenario indeed indicates a potential slight increase in hospitalization in 2021 (range: -6 to +2%) but not for the 2021-2022 period (range : -18 to -21%) and not for all other scenarios. This rather counterintuitive result stems from the fact that the vaccination program indirectly impacts the level of NPI in our analysis which remains an important driver for the overall COVID-19 burden in 2021 (lower NPIs are associated with a larger number of hospitalizations). As it corresponds to a rather extreme scenario and is no longer observed when a more adequate time horizon for assessing vaccination benefits is considered (i.e. 2021-2022), this result should not be overstated. Nevertheless, it reinforces the message in our manuscript around the need for a combination of vaccination and NPI in 2021.

**In continuation of the above, the authors should explore the solution space in much greater detail. They should clearly identify the scenarios where the outbreak ends in a reasonable time-frame instead of continuing on into 2023 and beyond. This should be used to motivate a discussion of effective vs ineffective vaccines and good vs bad policy decisions during the vaccination drive.**

🡪 We focused our scenarios in the next 2 years due to the substantial uncertainties of a long-term modelling. The increase seen post-2022 is driven by the assumption that both natural and vaccine induced immunity last on average 1 year. This assumption comes from (population based and challenge) studies of human coronaviruses in the 80s and later. There are yet limited data to support long lasting assumptions (i.e., longer than 1 year). This is particularly relevant as we consider changes in the human antibody immune response to variant viruses already circulating. We have added this discussion to the manuscript more explicitly and indicated how the various constraint scenarios can affect the impact of vaccination in curtailing the pandemic effect, addressing reviewer’s concern.

**Apart from the above major concerns, there are several minor issues as well, as given below.
5 The population of France should be mentioned so that the supply constraints can be understood in terms of percentage population.**

🡪 the percentage coverage of the total population for all scenarios are presented in table 1, as well as for the subgroups. We added a reference to the total population for clarity as suggested.

**6 The introduction should be updated to reflect the current situation of vaccination drives. EUA granted to Pfizer, Moderna, Oxford/ Astra Zeneca, ICMR/ Bharat Biotech and Sputnik vaccines should be mentioned.**

🡪We have updated the text to reflect advances in the pipeline, the authorizations as well as implementation to date.

**7 In Tables 1 and 2, the phrases “relaxed strong/weak supply and uptake constraint” is not clear to me. By relaxing a constraint one typically understands that the constraint does not exist any longer. However this does not seem to be what the authors imply.**

🡪 (similar response to reviewer 1): we have five scenarios of varying constraints. The terminology aims to reflect the programmatic evaluation literature and we want to highlight the limitation of programs as well as the dynamic aspect of the limitations. To clarify, we have added the following explanations to table 1.

- Uptake constraint reflects a limited coverage rate due to a limited willingness to be vaccinated in the population.
- Strong supply constraint reflects a limited amount of vaccines doses made available to the national program. In this case, the program is severely limited.
- Weak supply constraint reflects a limited amount of vaccines doses made available to the national program, in this case the program is moderately limited.
- Relaxed strong supply and uptake constraints. In this scenario, while the program is severely limited during the first half of the year, vaccine supply is increased during the second half of the year (higher production or new vaccines availability) and the public is more likely to be willing to vaccinate as the program has been in place for half a year. Therefore, we ‘eased’ the limitations of the program in the second semester.
- Relaxed weak supply and uptake constraints. In this scenario, while the program is moderately limited during the first half of the year, vaccine supply is increased during the second half of the year and the public is even more likely to be willing to vaccinate as the program has been in place for half a year. Therefore, we ‘eased’ the limitations of the program to achieve maximum coverage.

**8 Figure 4 is barely legible; moreover, it is difficult to understand the point attempted to be conveyed by the authors. The authors must improve the clarity of presentation here.**

- As there is still a large number of uncertainties on COVID-19 evolution, we used a tornado diagram approach for assessing separately the potential impact of important uncertainty factors that is presented Figure 4.
  To facilitate the readability of this figure, in the revised version, we only kept in the main text the panel A (univariate sensitivity analysis on reduction over the 2021-2022 period) and added panel B to supplementary material). In response to the comment on duration of immunity, we also added a sensitivity analysis on this topic.

**9 Factors like governmental support have not been considered.**

🡪 Governments have supported vaccine development by providing push and pull incentives – these include financing of R&D and production at risk as well as the streamlining of regulatory processes. We touch upon these in the introduction. Once the vaccine has been developed, in France, the design and implementation of the immunization program is government driven and thus, it is intrinsically supported by the national public health and health systems. We added some background around this narrative to the program description.

**10 Currently there are several viral strains such as B1.1.7 and B1.351 that might interfere with the effectiveness of vaccinations. It would be really impactful if the authors would mention about these variants as well.**

🡪 We agree with the reviewer that the emergence of new variants with reported increased transmissibility and/or severity and threats for vaccine-induced immunity is a critical factor for the future evolution of COVID-19. However, the evidence gathered around these new variants is very recent and a complete analysis of their consequences is beyond the scope of our manuscript.
In this revised version, we added the discussion a paragraph of the potential consequences of these new variants that actually reinforces our conclusion around the need for NPIs alongside vaccination

**11 Another important variable is the availability of required man-power which was not mentioned in the paper which might influence the supply and uptake constraints.**

🡪 The programs we modelled included assumptions on the ability of the health system to scale up, especially in the first half of 2021. The rate of vaccination is influenced by the availability of human resources, building space and consumables among others. We added this explanation to the methods.

**12 Although it is a modelling study, but as the core topic was vaccination, some concepts on involvement of antibodies or other physiological concepts could have made the story more interesting. Not required though.**

🡪 Thank you for your comment. Although we agree with the reviewer, it is difficult to extrapolate and expand on aspects of immunity in a manuscript that used modeling to discuss uncertainties related to the evolution of the pandemic in a broad population sense. However, we have added to the discussion on the aspects of vaccine response to give more depth to the paper.

**In summary, the Article as written presents a very strong claim without basing it on a sufficiently solid foundation.**

🡪 our conclusion is that a limited immunization program may not be sufficient in the short term to avoid further spread of SARS-CoV-2. If the constraints of such an immunization program are addressed in the second half of the year, the impact increases and more control over the epidemic is realized. With this conclusion we aim to highlight the importance of considering vaccination impact in realistic contexts which includes implementation and supply constraints and vaccine hesitancy in the short term. We hope with the additional text included in the methods, results and discussion we have addressed this concern.

**It also suffers from avoidable defects of presentation.**

🡪 we have addressed these defects as well as issues with the reference list. Thank you

**Hence I recommend the authors to revise the manuscript along the lines indicated above and resubmit the revised version**

🡪 To the best of our ability we have addressed both reviewers’ comments. Thank you
