# Supplementary material for: Potential impact of introducing vaccines against COVID-19 under supply and uptake constraints in France: A modelling study
Source: PLoS One. 2021 Apr 28;16(4):e0250797. doi: 10.1371/journal.pone.0250797 (PMC8081204; doi:10.1371/journal.pone.0250797)
Supplement: Attachment — Submitted filename: Decision Letter.pdf [file pone.0250797.s002.pdf]

Manuscript ID : PONE-D20-37719

Title : Potential impact of introducing vaccines against COVID-19 under supply and uptake constraints in France : a modeling study

Authors : Coudeville L et. al.

In this manuscript the authors present an analysis of the COVID-19 spreading dynamics in France after the initiation of vaccination drive. The incorporation of constraints from both the supply and demand side adds realism to the model. Some statements from the results section drew my attention however. They are : (*a*) that even with the uptake constraint i.e. maximal vaccine distribution, there would be NPIs being applied in 2022, (*b*) that with discontinuation of NPI at the end of 2021, there would be +0·8 percent, -9·4 percent and (presumably negative although this has not been indicated by authors) 13·9 percent variation in hospitalizations in 2021 with the three vaccine constraints relative to the no vaccine case, and (*c*) that the incidence of COVID-19 remains significant at the end of 2022 with all situations of vaccination and even on the rise for most of them. These statements are counter-intuitive and a bit pessimistic – most people are hoping for a return to normalcy by this fall or at least by the end of the year. I would like the authors to recognize and discuss this fact in detail.

The authors could consider several major and minor revisions. Some major revisions are given below:

1 There are certain modeling studies of vaccination dynamics which present a more optimistic view than the authors' work. A key example is

Shayak B, Sharma MM and Mishra AK, "Impact of immediate and preferential relaxation of social and travel restrictions for vaccinated people on the spreading dynamics of COVID-19 : a model-based analysis," available at

https://www.medrxiv.org/content/10.1101/2021.01.19.21250100v1

The references by Alvarez et. al. and Betti et. al. in the above manuscript are also optimistic. While I am aware that all these works were written after the authors' manuscript, they must now be cited. It must be explained why the authors' results differ from these analyses.

2 What is the role of the temporary immunity in generating the authors' case trajectories ? In other words, if the vaccine immunity had been 2 years or 5 years, then what would the trajectories have been like ? Authors should perform simulations to demonstrate this.

3 What is the role of the bang-bang NPI control strategy in generating the authors' bleak predictions ? Instead of this strategy, if a continuous NPI were applied or NPI gradually relaxed over time then what would the trajectories have looked like ? Simulations should be performed to analyse these questions.

4 Why there is an *increase* in hospitalization in 2021 with strong supply constraint relative to no vaccination ? Surely this is a surprising result. In continuation of the above, the authors should explore the solution space in much greater detail. They should clearly identify the

scenarios where the outbreak ends in a reasonable time-frame instead of continuing on into 2023 and beyond. This should be used to motivate a discussion of effective vs ineffective vaccines and good vs bad policy decisions during the vaccination drive.

Apart from the above major concerns, there are several minor issues as well, as given below.

5 The population of France should be mentioned so that the supply constraints can be understood in terms of percentage population.

6 The introduction should be updated to reflect the current situation of vaccination drives. EUA granted to Pfizer, Moderna, Oxford/ Astra Zeneca, ICMR/ Bharat Biotech and Sputnik vaccines should be mentioned.

7 In Tables 1 and 2, the phrases "relaxed strong/weak supply and uptake constraint" is not clear to me. By relaxing a constraint one typically understands that the constraint does not exist any longer. However this does not seem to be what the authors imply.

8 Figure 4 is barely legible; moreover, it is difficult to understand the point attempted to be conveyed by the authors. The authors must improve the clarity of presentation here.

9 Factors like governmental support have not been considered.

10 Currently there are several viral strains such as B1.1.7 and B1.351 that might interfere with the effectiveness of vaccinations. It would be really impactful if the authors would mention about these variants as well.

11 Another important variable is the availability of required man-power which was not mentioned in the paper which might influence the supply and uptake constraints.

12 Altough it is a modelling study, but as the core topic was vaccination, some concepts on involvement of antibodies or other physiological concepts could have made the story more interesting. Not required though.

In summary, the Article as written presents a very strong claim without basing it on a sufficiently solid foundation. It also suffers from avoidable defects of presentation. Hence I recommend the authors to **revise** the manuscript along the lines indicated above and **resubmit** the revised version.
